# High prevalence of urinary schistosomiasis in two communities in South Darfur: implication for interventions

**DOI:** 10.1186/1756-3305-4-14

**Published:** 2011-02-07

**Authors:** Kebede Deribe, Abdeljbar Eldaw, Samir Hadziabduli, Emmanuel Kailie, Mohamed D Omer, Alam E Mohammed, Tanole Jamshed, Elmonshawe A Mohammed, Ali Mergani, Gafar A Ali, Khalid Babikir, Abdulrahman Adem, Farouq Hashim

**Affiliations:** 1 American Refugee Committee International, Nyala, South Darfur, Sudan; 2 State Ministry of health, Nyala, South Darfur state, Sudan; 3 World Health Organization WHO South Darfur Sub Office Nyala, Sudan; 4 UNICEF Nyala Office, South Darfur, Sudan; 5 State Ministry of Health, Department of Epidemiology, Nyala, South Darfur, Sudan; 6 State Ministry of Health; Malaria, Schistosomiasis and Leishmaniasis control program, Nyala, South Darfur, Sudan

## Abstract

**Background:**

There are few data on the prevalence of schistosomiasis in Darfur. We conducted this study in response to reports of 15 laboratory confirmed cases of schistosomiasis and visible haematuria among children from two communities in South Darfur. The aim of the study was to estimate the prevalence of schistosomiasis in the area and to decide on modalities of intervention.

**Methods:**

A cross-sectional survey involving 811 children and adults from schools and health facilities was conducted in two communities of South Darfur in March 2010. Urine samples were collected and examined for ova of *Schistosoma haematobium *using a sedimentation technique. A semi-structured format was used to collect socio-demographic characteristics of the participants.

**Results:**

Eight hundred eleven (811) urine samples were collected, 415 from Alsafia and 396 from Abuselala. Of the collected samples in 56.0% (95% Confidence Interval (CI); 52.6-59.4) *Schistosoma *eggs were found. The prevalence was high in both Abuselala 73.3% (95% CI; 68.9-77.6) and Alsafia 39.5% (95% CI; 34.8-44.2). More males (61.7%, 95%CI; 56.5-64.9) were infected than females (52.1%, 95%CI; 48.2-56.0). Children in the age group 10-14 has the highest (73.0%, 95%CI; 68.7-77.2) infection rate. School age children (6-15 years) are more likely to be infected than those >15 years (Adjusted Odds Ratio (AOR) = 2.70, 95% CI; 1.80-4.06). Individuals in Abuselala are more likely to be infected than those who live in Alsafia (AOR = 4.3, 95% CI; 3.2-5.9).

**Conclusion:**

The findings of this study indicate that *S. hematobium *is endemic in Alsafia and Abuselala South Darfur in Sudan with a high prevalence of infection among older children. This signifies the importance of urgent intervention through Mass Drug Administration (MDA) to halt the infection cycle and tailored health messages to targeted groups. Based on the findings MDA was conducted in the villages.

## Introduction

Schistosomiasis or bilharzia is a tropical parasitic disease caused by blood-dwelling flukes of the genus *Schistosoma *[1]. It affects about 200 million people worldwide, and more than 650 million people live in endemic areas [1,2]. Urinary schistosomiasis is caused by *Schistosoma haematobium *and is endemic in sub-Saharan Africa, including Sudan. Estimates for this region of the world suggest that 436 million people live at risk of infection with *Schistosoma haematobium *[3,4].

Although schistosomiasis is highly prevalent, the associated morbidity is low and variable [1]. However, the Global Burden of Disease Study, attributes a disability weight of 0·06 and an annual mortality of 14 000 deaths per year to schistosomiasis. Based on 200 million infected people worldwide, the total number of Disability Adjusted Life Years (DALY) lost to schistosomiasis is estimated at 1·532 million per year, of which 77% are in sub-Saharan Africa [5]. New meta-analyses indicated a mortality estimate up to 280,000 deaths annually in sub-Saharan Africa alone [4,6].

Although the intimate connection between conflict and Neglected Tropical Disease (NTD) such as schistosomiasis is known [7], the prevention and control of such disease in conflict and emergency settings are often neglected. In such contexts, more emphasis is given for lifesaving services and diseases of epidemic importance. If the prevention and control of such diseases do occur, they are often uncoordinated and driven by the occurrence of a cluster of cases.

American Refugee Committee International (ARC) has been working in Darfur since October 2004. This study was conducted in response to American Refugee Committee's (ARC) report of cases of schistosomiasis in Abuselal and Alsafia to State Ministry of Health (SMoH). Fifteen laboratory confirmed cases of schistosomiasis from Alsafia and visible haematuria among children from Abuselala were reported through ARC supported health facilities. Therefore this study was conducted to estimate the prevalence of schistosomiasis in the area and to decide on modalities of intervention.

## Materials and methods

### Study site

The survey was conducted in two villages in South Darfur State, south western region of North Sudan during March 2010. The villages (Alsafia and Abuselala) are located 75 and 103 Kilometers Southwest of Nyala the capital of the state. The villages lies between Latitude N: 11 12 52.95 and Longitiude: E: 24 42 47.29. The villages have an estimated population of 5,000 (Abuselala) and 9,000 (Alsafia). The State experiences a single rainy season, typically between June and October. The villages have seasonal ponds, the inhabitants utilize the ponds for fishing, domestic purposes, and children and young adolescents swim regularly in the ponds[8]. Both areas have no history of treatment for schistosomiasis and this study is the first of its kind in this setting.

### Sample population and selection

For schistosomiasis control, WHO recommends that 200-250 school-aged children sampled in each ecological zone [9]. Children in school, Khalwas (religious schools), and children and adults in health facilities were included in the study. The data was collected from 811(415 Alsafia and 396 Abuselala) individuals. A total of 323 and 329 school-age chidren were enrolled in the study from Abuselala and Alsafia respectively, which is more than the WHO minumum recomendation. In addition adults and under-five children were enrolled to see the pattern of infection acrosse age groups.

### Data collection

In brief, for all individuals, age, sex and location were captured through a semi-structured format. Mass urine investigation of schistosome eggs identification was conducted. Parasitological examination of urine samples was conducted in the field by a team of trained laboratory technicians. From each subject, a 10 ml sample of terminally voided urine was collected in a properly labeled clean and sterile specimen container between 10.00 and 14.00/h. Centrifugation and sedimentation techniques [10] were employed to analyze the samples. 10 ml urine was taken from the deposit of each specimen bottle after allowing to sediment for about an hour and centrifuged for 2 mins at 2000 rpm. The deposit was thereafter examined microscopically using ×10 and ×40 objectives for the characteristics schistosome egg. Urine samples containing egg(s) of *S. haematobium *were recorded as positive, while absence of eggs of schistosomes was considered as negative [10].

### Data management and statistical methods

Data were edited, cleaned, and analyzed using SPSS for Windows version 16.0 (SPSS Inc., Chicago, IL, USA). To calculate the prevalence, descriptive analysis was done. Bivariate and multivariate logistic regression analysis was done to examine the association between socio-demographic variables and schistosomiasis. A simple regression model was produced to identify factors associated with infection by adjusting for age, sex and residence.

### Ethical considerations

The study was approved by SMoH, followed by the Local Health Authority. The study was explained to each participant. Individuals or caregivers were asked to consent verbally to participate in the study; only those who provided consent were registered and requested to provide samples. To document verbal consent, the name of each individual who provided verbal consent was recorded, along with the test results for their samples. Individuals who tested positive for schistosomiasis infection were treated with praziquantel, according to WHO guidelines. Due to shortage of medicine some (52) of the participants were referred to health facilities. Parents/guardians provided consent on behalf of all child participants.

## Results

### Socio-demographic characteristics

In this study 811 individuals were involved, 415 from Alsafia and 396 from Abuselala. Most (84.5%) of the participants were in primary school age group. The median age was 10 (inter-quartile range (IQR): 8-13 years). The majority (54.1%) of the participants were male; the socio-demographic characteristic of the participants is shown in Table 1.

**Table 1:** Sociodemographic characteristics of study participants South Darfur, March 2010.

Variable	Category	Number	Percent
Age in years	≤5	15	1.8
	6-15	652	80.4
	>15	144	17.8
Address	Abuselala PHC	193	23.8
	Abuselala School	203	25.0
	Alsafia all	415	51.2
Sex	Male	445	54.9
	Female	366	45.1

### Prevalence of urinary schistosomiasis

Urine samples were taken from different sample groups; students from Abuselala School, patients and clients in Abuselala PHC and in and out of school children and adults in Alsafia. In total, 454/811 (56.0%, 95% Confidence Interval (CI); 52.6-59.4) individuals harbored *Schistosoma *eggs. The overall prevalence among school age (6-15 years) children is 60.6% (76.8% in Abuselala and 44.7% in Alsafia). The prevalence was significantly high (73.3%, 95% CI; 68.9-77.6]) in Abuselala compared to Alsafia (39.5%, 95% CI; 34.8-44.2) (p < 0.001). Similarly the prevalence was significantly high (60.6%) among children in 6-15 years age, followed by >15 years (38.2%) and ≤5 children (26.7%) (p < 0.001). Likewise the prevalence was significantly high (61.7%, 95CI; 56.5-64.9) among male compared with female (52.1%, 95%CI; 48.2-56.0) (p = 0.015). In a separate analysis, children in age groups 10-14 and 15-19 were more likely to be infected than children in the age group 5-9 with odds of (OR = 2.90, 95% CI; 2.08-4.06) and (OR = 2.42, 95% CI, 1.37-4.28) respectively.

### Factors associated with presence of Schistosoma eggs

All the factors (age, sex and address) associated with presence of Schistosoma eggs were entered into logistic regression model and only age and address were independently associated. Children in the age range of 6-15 are more likely to be infected than those >15 years old (OR = 2.70, 95% CI; 1.80-4.06). Likewise those individuals who live in Abuselala were more likely to be infected than those who live in Alsafia (OR = 4.31, 95% CI, 3.17-5.86). (Table 2)

**Table 2:** Prevalence of Schistosomiasis among study participants South Darfur, March 2010.

Variables	Test Result		COR(95%CI)	AOR(95%CI)	P-value
			
	Negative	Positive			
Age in years					<0.001
≤5	11(73.3%)	4(26.7%)	0.59(0.18-1.94)	0.69(0.20-2.43)	
6-15	257(39.4%)	395(60.6%)	2.49(1.72-3.60)	2.70(1.80-4.06)	
>15	89(61.8%)	55(38.2%)	1.0	1.0	
Address					<0.001
Alsafia	251(60.5%)	164(39.5%)	1.0	1.0	
Abuselala	106(26.8%)	290(73.2%)	4.18(3.11-5.64)	4.31(3.17-5.86)	
Sex					>0.05
Male	144(39.3%)	222(60.7%)	1.42(1.01-1.87)	1.02(0.74-1.39)	
Female	213(47.9%)	232(52.1%)	1.0	1.0	

*Adjusted for age, sex and address

## Discussion

The results of this study show a prevalence rate of 56% of urinary schistosomiasis among the respondents in Darfu. The result is higher than reported in previous studies in the state [11,12].

*S. haematobium *has previously been reported in Southern Darfur in earlier years [13]. The snail intermediate host *Bulinus truncatus *is generally found in Southern Darfur where it is well adapted to pools and slow-flowing waters [14].

In Sudan the risk for schistosomiasis is widespread, especially in the major irrigation systems in the Gezira area between the Blue and White Nile Rivers - high prevalence of *S. mansoni *infection in some areas in the West Equatoria region and both *S. mansoni *and *S. hematobium *are highly endemic in the Upper Nile region. In the country 5 million people are infected by schistosomiasis [15,16].

The higher prevalence rate of urinary schistosomiasis in this study reflects the higher level of exposure and dependence of the inhabitants on the infected water bodies. There are small ponds in the areas which are used for fishing, household utilization and swimming. Similar to other studies [17,18] the prevalence of schistosomiasis is high among children in age group 10-14. The disparity in the pattern of the infection among the individuals of different age groups with school-age children having the highest infection rate and the least inflections occurring among children in pre-school age could be due to low exposure. Among the children, the females had relatively lower infection than their male counterparts. This could be due to difference in exposure status. In a separate analysis there was no significant difference in the rate of infection observed among adult females and males. Respondents in Abuselala have higher infection rate than those found in Alsafia and this could be attributed to types of water bodies and water contact practices which need further investigation.

This study has a number of limitations; therefore the findings should be interpreted in the light of these limitations. First of all, we have not collected data on environmental and lifestyles of the participant which might have implication on interpretation of the data and implication for intervention. Secondly, we did not measure intensity of the infection. Thirdly, we have included individuals attending health facility which might overestimate the prevalence of the infection. Finally we used a sedimentation method which, although recommended by WHO is less sensitive and might underestimate the prevalence.

In light of the above limitation the findings of this study suggest that *S. hematobium *is endemic in Alsafia and Abuselala South Darfur in Sudan with a high prevalence of infection among older children and affecting more male than female. In complex emergencies, health services provision usually focuses on epidemic important diseases and life saving services but often ignores endemic diseases. Although in the initial phases of an emergency, interventions should focus on the most critical diseases. In the later stages, integrated approaches towards treating tropical disease and local endemic disease should be considered.

### Implication for intervention

The data on the prevalence of urinary schistosomiasis, where 56% of the volunteers examined harbored *S. haematobium *ova in their urine, indicated endemicity of the infection. World Health Organization categorizes level of infection based on prevalence among school age children. The guideline recommends that, in communities with schistosomiasis, and a prevalence of 10% up to 50%, school-aged children and high-risk groups of adults should be treated with praziquantel once every two years. In communities where prevalence is 50% and above, the same groups should be treated once a year [2]. The prevalence of schistosomiasis among school-age children (76.8%) as evidenced by *S. haematobium *ova in urine, indicated that Abuselala exceeds this Mass Drug Administration (MDA) threshold and qualifies for annual MDA. In Alsafia the prevalence is 44.7% among school age children and thus merits MDA every two years (Table 3).

**Table 3:** Pattern of prevalence rates of *S. haematobium *among different age groups South Darfur, March 2010.

Age group	Negative	Positive
0-4	11(73.3%)	4(26.7%)
5-9	171(51.8%)	159(48.2%)
10-14	80(27.0%)	216(73.0%)
15-19	20(30.8%)	45(69.2%)
20-24	21(67.7%)	10(32.3%)
25-29	22(64.7%)	12(35.3%)
30-34	10(76.9%)	3(23.1%)
35-39	7(58.3%)	5(41.7%)
> = 40	15(100.0%)	0(0.0%)

Tailored health education for the community is required for the control and prevention of urinary schistosomiasis and its attendant illnesses in the study areas [1,19,20]. Inclusion of schistosomiasis in the primary school curriculum would help to address children in the school. Finally, MDA should not be a substitute for implementation through regular health services. Strengthening the health system and building the capacity of health workers in diagnosis and treatment of schistosomiasis remains the only option to address those ineligible for MDA.

### Interventions

The result of the survey was presented in the weekly health coordination meetings with the resource required to conduct the intervention. Based on the request, multiple agencies committed resources for the MDA. Before the mass treatment, meetings were organized with community leaders and health education was provided through community health workers. The dosage of praziquantel was 40 mg/kg body weight. Praziquantel tablets were procured through the UNICEF Supply Division in Copenhagen, Denmark and shipped to Sudan. Individuals at least 5 years old were eligible for treatment. Exclusion criteria included reported hypertension measured on site, self-reported history of asthma, allergy, pregnancy, lactation, recent delivery, possible pregnancy, seizure disorder, past surgery or currently febrile state. The provision of praziquantel adhered to the Ministry of Health guidelines. Body weight was recorded and consumption of doses of praziquantel was directly observed by drug distributors. A total of 13,469 individuals received a dose of praziquantel.

## Competing interests

The authors declare that they have no competing interests.

## Authors' contributions

KD, GAA, KB, AA, AM designed the study. SH, FH, AEM, MDO assisted in data collection and analysis. TJ, EAM involved in designing the study and data collection. KD, AE, EK analyzed the data and wrote the first draft of the manuscript. All authors contributed to the manuscript and approved its final version.
